# The University of Buffalo

**Published:** 1905-06

**Authors:** 


BUFFALO MEDICAL JOURNAL.

A Monthly Review of Medicine and Surgery.

EDITOR:

WILLIAM WARREN POTTER, M. D.

All communications, whether of a literary or business nature, books for review and exchanges, should be addressed to the editor 284 FRANKLIN ST., BUFFALO, N. Y.

VOL. XLIV.—LX. JUNE, 1905. NO. 11

### The University of Buffalo.

ELSEWHERE we publish in considerable detail the proceedings of commencement week at the university, including the work and play of the alumni association. There are, however, some things which took place not included in our report or are mentioned only incidentally, and which we desire to elaborate or accentuate in this place.

To begin with, it should be noted that the alumni proceedings occupied two days, the most of which time was devoted to clinics at the several hospitals and in the university amphitheaters. This experiment proved successful in general and only imperfect in minor particulars incident to all innovations. If any mistake was made it was in attempting to accomplish too much in the narrow limit of time at disposal. In our opinion it would be wise another year to extend the meeting to include an entire week with clinics morning and afternoon, scheduled in such a way that the entire body may attend each clinic if desirable. In other words, we think it would be well to utilise all the available part of commencement week in a way that would give it the character of a postgraduate course. In this way the attendance at the alumni meeting would be increased we believe; or, if not, the college would derive an indirect benefit from the attention attracted to it by this quasi postgraduate term. This is a subject that deserves to be carefully matured and, if adopted, definitely announced for a sufficient period beforehand, to enable the alumni residing at a distance to make plans for attendance.

Another important feature relating to the welfare of the university was the announcement made by Professor Mann, at the evening session of the alumni association, that the term would be increased to eight months and the teaching day shortened one hour. The addition of a month to the term is wise action and the shortening of the lecture day relieves the students of overwork and especially of overstrain. The increase in marking percentage to 75 as a minimum also is in the line of progress.

It was also announced by Dr. Mann that in future the BUFFALO MEDICAL JOURNAL would be in closer touch with the university than had been the case for some time past. There is every reason to expect, he continued, that the entente cordiale reëstablished would prove to the mutual advantage of both Journal and university. The arrangement made with the editor, he said, did not change in any way the management of the Journal, but it did mean that the alumni, if loyal to the university, would also lend support to the Journal by contributions of papers and subscriptions. The editor of the Journal in his remarks at the alumni banquet, made further reference to the restored community of interest between the university and the Journal.

One of the absorbing questions relating to university interests is the establishment of a department of letters or liberal arts. This has been discussed at various times and again has been dropped as being inexpedient. Now, however, the time seems ripe for fighting the campaign to a successful conclusion. The alumni of the several large universities in the United States, residing in this city, have organised under the title of the associated Alumni of Buffalo, and are making an aggressive canvass relating to university extension. Committees have been appointed to investigate various phases of the proposition, which are named elsewhere. The president of the medical alumni association, Dr. DeLancey Rochester, also appointed a committee from that body to coöperate with the others, the names of which are D. W. Harrington, '71, chairman, and Thomas H. McKee, '98, of Buffalo; and W. R. Campbell, '80, of Niagara Falls.

Vice-chancellor Norton made university extension the burden of his speech at the alumni banquet, the substance of which we publish in another part of the Journal and to which we invite most considerate attention. The outlook for a consummation of this devoutly to be desired expansion of the university equipment seems favorable. Let every person attached to the teaching body of the department of medicine manifest an interest in its success. Every individual holding relationship to the college and hospitals should make it a paramount duty to attend the alumni association meetings, and thus testify to a personal zeal in the success of everything that pertains to building up a strong university in the city of Buffalo.

One more thought: it would seem to us a good plan to make the commencement exercises of the department of law coincident with the other departments. If all graduation ceremonies were held at the same time and place it would tend to consolidate interests and to concentrate energy.

We make no apology for the abundant space we have appropriated elsewhere in reporting the college and alumni proceedings; it is a subject in which every Buffalo physician as well as every alumnus of the university should take interest.

